# Why is income volatility associated with poor health? Longitudinal evidence from the UK and France

**DOI:** 10.1016/j.ssmph.2025.101869

**Published:** 2025-10-06

**Authors:** Daniel Nettle, Coralie Chevallier, Kate E. Pickett, Matthew T. Johnson, Elliott A. Johnson, Melissa Bateson

**Affiliations:** a Département D’études Cognitives, Ecole Normale Supérieure, Institut Jean Nicod, Université PSL, EHESS, CNRS, Paris, France; b Department of Social Work, Education and Community Wellbeing, Northumbria University, Newcastle Upon Tyne, UK; c Department of Health Sciences, University of York, York, UK; d Biosciences Institute, Newcastle University, Newcastle Upon Tyne, UK

**Keywords:** Income volatility, Self-rated health, Anxiety, Depression, Socioeconomic gradients

## Abstract

There is some evidence that income volatility (fluctuations in income over time) negatively affects mental and physical health, independently of the level of income. Evidence to date has examined fluctuations from year to year or from day to day, whereas a more relevant timescale might be month to month. Here, we use data from the Changing Cost of Living Study, a longitudinal panel from the UK and France with monthly data (n = 484). We examine the association between month-to-month income volatility and two outcomes, self-rated general health and anxiety-depression (a composite measure derived from GAD-7 and PHQ-8 scores). Higher volatility was associated with worse health on both measures, with volatility accounting for similar amounts of variation as the level of income. Some association between income volatility and health is to be expected as a consequence of the concavity of the income-health relationship: because of concavity, a downward fluctuation damages health more than the equivalent upward fluctuation improves it. We show that the observed associations are 3 and 4 times too strong to be explained by this mechanism alone. We suggest that volatility, because it introduces uncertainty and stress, has substantial direct health effects. This claim is important for public policy: it means that policies and institutions that smooth people's income streams can have beneficial health effects even if they don't raise anyone's income.

## Highlights

• Monthly income fluctuations were associated with poorer health, more anxiety and depression.
• The associations were too strong to be due to concavity effects alone.
• Income volatility may in itself harm physical and mental health.
• Policy interventions that smooth income flows could be positive for population health.

## 1 Introduction

There is increasing evidence that volatility of income is associated with poorer mental and physical health, even after adjusting for the person's level of income (Adeline et al., 2019; Grasset et al., 2019; Rohde et al., 2016; Sayre, 2023). This has important implications for population health, because income volatility is common. In the UK, for example, only 26 % of employees receive an income that falls within plus or minus 10 % of their personal average in every month of the year (Unstable pay resolution foundation, 2025). High volatility is most common amongst those with the lowest incomes, working part-time, and on temporary contracts. It is very prevalent in sectors relying on tips and commissions, where shift patterns are variable, and the ‘gig’ economy (Sayre, 2023). The studies of volatility and health use time series of incomes from the same individuals, and operationalize volatility as the variation or standard deviation of the time series or some related metric. To date, they have focused on day-to-day volatility, for people in tipped or erratic occupations (Sayre, 2023), or year to year variation for the general population (Adeline et al., 2019; Rohde et al., 2016), because administrative data and many panel studies record annual incomes. This is a limitation, since critical household expenditures such as rent and utility bills often fall in a monthly cycle rather than a daily or annual one. Month-to-month volatility, the volatility we study here, would seem particularly relevant to people's ability to meet basic needs, and hence their health.

**Fig. 1:**
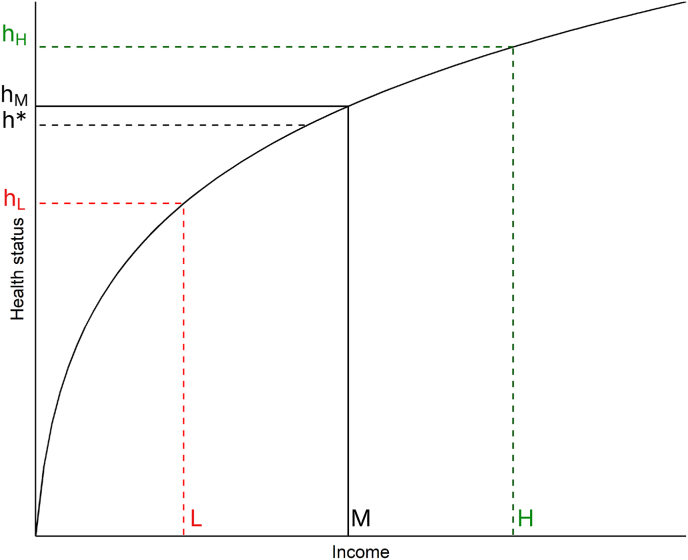
Illustration of why greater income volatility must necessarily produce worse health over time. Compare two individuals with the same mean income M. One earns M every month, whilst the other alternates between L and H. Given the relationship between income and health status depicted in the curve, the health of the individual with the constant income will be h_M_, whilst the health status of the individual with the volatile income will half the time be h_H_ and half the time h_L_. Because of the concavity, the average of h_H_ and h_L_ (which is depicted h∗) must be lower than h_M_.

Research to date has left a theoretical question unanswered. Given that the relationship between income and health is known to be concave (Backlund et al., 1996; Nettle & Dickins, 2022; Rehnberg & Fritzell, 2016), it is mathematically inevitable that, of two people with the same average level of income but where one has greater volatility, the average health status over time will be worse for the one with greater volatility (see Fig. 1 for explanation). This is because the worse-than-average months will be more detrimental to health than the better-than-average months will be beneficial to it. This is known as a concavity effect. Concavity effects are well understood in the study of the relationship between between-individual income inequality and population health, where they lead to average health being poorer in populations where incomes are more dispersed (Gravelle, 1998; Rodgers, 1979). Income volatility can be thought of as within-individual inequality in income, and thus the same statistical arguments apply as for between-person inequality.

The outstanding theoretical question for the study of income volatility and health is thus the following. Is the observed association between higher income volatility and worse health entirely due to concavity effects as shown in Fig. 1, or is there an additional health-detrimental effect of the volatility itself? If the concavity effects illustrated in Fig. 1 were the complete explanation, the health of a person who alternates between an income of L and an income of H would be the average between the health and a person whose income is always L, and the health and a person whose income is always H. There would be no additional health cost of the volatility *per se*.

However, it is plausible that there is such a volatility cost. There are many possible reasons for this. Income volatility could have negative practical consequences, and also generate uncertainty. Uncertainty is closely linked to stress (Berker et al., 2016; Peters et al., 2017). Authors of the existing papers on income volatility and health have suggested that income volatility is in itself damaging for health, but their analyses have only shown that the association exists, not shown that it is stronger than can be explained by concavity effects alone. Thus, it may not be necessary to invoke additional mechanisms based on uncertainty and stress due to volatility. One of the central aims of this paper is, therefore, to establish whether associations between income volatility are only as strong as follows from concavity effects, or are stronger than this. This question is of considerable policy relevance. For example, if there is a stronger association between volatility and ill health than can be attributed to concavity alone, then economic reforms that smooth people's income flows will have a beneficial effect on population health even if they do not increase anyone's average income.

This paper addresses the relationship between income volatility and health (self-rated general health, and a measure on mental health based on widely-used scales of anxiety and depression symptoms) in the Changing Cost of Living Study (CCLS). This was an intensively longitudinal study of small panels in the UK (n = 244) and France (n = 240). The panels were not nationally representative. They were opportunity samples recruited via research participation agencies in the two countries; although there were no selection criteria other than age of 25 or over, participants tended to be concentrated at the lower end of the income distribution (see Nettle et al., 2024 for full description). Participants reported, once a month for a year, the income received, expenditures on essential costs, and a range of psychological and health measures, including self-rated general health on a 1–9 scale, and current symptoms of anxiety (the GAD-7 scale; Spitzer et al., 2006) and depression (the PHQ-8 scale; Kroenke et al., 2009). The CCLS is uniquely suited to addressing the present question, since the monthly time scale of the measurement points correspond to the timescale over which fluctuations most impact people, because key bills and payments are likely to fall on a monthly cycle.

The research questions for the study were as follows:

1. Does higher volatility in income predict worse self-rated and mental health over the course of the study, after adjusting for level of income?
2. How strong an association between volatility and health should be observed as a product of concavity effects alone?
3. Is the association observed in 1, if present, stronger than would be expected due to concavity effects, and, if so, by what margin?
4. By how much would we predict self-rated and mental health to improve if income volatility were eliminated?

## 2 Methods

### 2.1 Participants and data source

We used the publicly available version 3 of the CCLS data (dataset and codebook at: https://osf.io/d9qb6/). Our analysis code is available at: https://osf.io/49w3z/. Participant panels were recruited in UK (n = 244) and France (n = 240) via online research participation platforms. Data were collected monthly, between September 2022 and August 2023. Respondents completed an average of 10 monthly reports each (s.d. 3). The panels were not nationally representative and were biased towards the lower end of the income distribution, especially in France (see Nettle et al., 2024 for further details).

### 2.2 Measures

Income was self-reported, as the number of pounds or euros actually received over the course of the month. Values in pounds were converted to euros. The CCLS dataset also contains measures of essential costs and income-to-costs ratios. Costs tend to fluctuate less than income from month to month and none of the conclusion of this paper would be affected by adjusting income for costs. Likewise, the dataset contains variables allowing incomes to be equivalised for household size; doing so does not affect the conclusions and we prefer to use the simple income variable.

Self-rated general health was reported on a scale of 1–9 (very bad - very good). Despite their simplicity, single-item self-rated health variables have been shown to have high validity and utility. For example, they predict mortality (Idler & Benyamini, 1997; Schnittker & Bacak, 2014), respond to disease onset and functional limitation (Wu & Zhang, 2023), and are appropriately associated with inflammatory biomarkers (Christian et al., 2011).

Our mental health measure (henceforth anxiety-depression) is a composite made from averaging the score on the GAD-7 scale (anxiety symptoms; Spitzer et al., 2006) and PHQ-8 scale (depression symptoms; Kroenke et al., 2009). We used the composite since the two variables were correlated at 0.86. Since the two scores have very similar means and standard deviations, they contribute about equally to the composite (GAD: mean 5.5, sd 5.37; PHQ: mean 6.01, sd 5.57). Similar results are obtained using either variable separately.

Note that anxiety-depression is a negative measure of health; higher scores indicate worse mental health. This means that the expected association with income is decreasing (higher incomes should be associated with lower scores); and the concavity that would produce concavity effects as discussed above is upwards concavity. This is in contrast to the self-rated health measure, where the expected association with income is positive overall, with concavity effects produced by downwards concavity.

### 2.3 Data analysis

We first aggregated data across the months, calculating for each participant their: average income; average self-rated health; and average anxiety-depression. We additionally calculated the volatility of income, operationalized as the standard deviation of the person's income values. This standard deviation will be larger the more volatile the person's income is. We note that the standard deviation would also be larger for someone who had a systematic upward or downward income trajectory over the year of the study, than for someone whose income had no temporal trend. We therefore also calculated a time-adjusted volatility statistic, which is the standard deviation of the residuals from a regression of income against time, fitted for each individual separately. This statistic captures, specifically, random shocks around the longer-term income trend. The simple standard deviation volatility statistic and the time-adjusted one were correlated at *r* = 0.93. All results were qualitatively similar using either. We therefore report results using the simple statistic.

Average income and income volatility were positively correlated (*r* = 0.35). This is to be expected since very low incomes cannot have a very large standard deviation in absolute terms. For this reason, we control for income itself in all models making inferences about the effect of volatility. Since coefficients in multiple-variable models are partial, this makes the quantity we are estimating - the impact of volatility for a given income - orthogonal to income itself.

An alternative to controlling for income in the models would be to express income volatility as the coefficient of variation (standard deviation divided by the mean). We avoid this approach, other than for visualizing relationships in Fig. 1. This is because, in order for the coefficient of variation in income to be statistically independent of income level, income variability would have to be strictly proportional to average income. This is far from the case: the regression of income volatility on average income has a non-zero intercept (0.24) and a slope much less than 1 (−71.53).

For research question 1, we fitted general linear models predicting self-rated and anxiety-depression from income plus income volatility. We also controlled for age and gender, since these are both well known to be independently associated mental health measures. Both income and income volatility were log transformed for this analysis.

We then adapted methods developed by Nettle and Dickins for addressing similar questions about between-person inequality (Nettle & Dickins, 2022). We used the month-level dataset to estimate the best-fitting relationship between income and the health variables, allowing for the relationship to be non-linear. We did this using Generalised Additive Models (GAMs). GAMs are semi-parametric models in which the outcome (here, the health variables) are related to a smooth function of the predictor (income) whose shape is determined by the data. The flexibility of the GAM approach (the shape of the smooth function is estimated from the data with many degrees of freedom) means that our models should capture the concavity of the income-health relationships very precisely. We used used the GAM models to predict the health scores that each person should have had in each month based solely on their income in that month. To answer research question 2, we averaged these scores across the months to obtain, for each person, the average health scores they should have been expected to have given their incomes over time and the observed concavity. To address research question 3, we compared the strength of association between income volatility and this concavity-predicted health with the strength of association between income volatility and the observed health variables.

We triangulated this analysis using a second method based on multilevel models. We used each month's report as a separate data point, with a random effect of participant. We entered income as a month-level variable, allowing for non-linearity using a GAM, and income volatility as a person-level variable. This approach provides a more direct inferential test of the extent to which income volatility matters above and beyond the concave impact of income itself, and we believe it to be the definitive analysis for research question 3. However, it is less intuitive and does not allow an easy decomposition of the overall associations of income volatility and health into the part attributable to concavity effects, and the part over and above concavity. Thus, we present it second in the paper.

GAMs were fitted in R packages ‘mgcv’ for the basic GAMs, and ‘gamm4’ for the multilevel models, using a thin-plate spine (the default, a flexible form of spine that does not require the number or location of knots to be pre-specified), with the smoothness parameter optimized via REML estimation. An alternative to the GAM approach is to fit a simpler parametric regression model that still allows for non-linearity. This is usually done by regressing the outcome variable on log(income). For comparison, we deploy this approach in the Supporting Materials (Appendix A). It leads to the same qualitative conclusions, though the association between income and concavity-predicted health is weaker using the logarithmic model, because that model captures the concavity present in the data less precisely than the GAMs.

To address research question 4, we used a counterfactual analysis. We used the regression models in which the health outcomes were predicted from income volatility, average income and control variables, to calculate, for each participant, the change in outcome that would be predicted if income volatility were 0 rather than its actual value. We were thus able to simulate the distribution of health outcomes under the counterfactual situation where every person had the same average income as they currently do, but with no volatility; and compare this to the actual health outcomes.

Although the average income and the averages of the health variables differ between the UK and French sub-samples of the data, there was no evidence that the relationships between income, income volatility and the health variables were any different. Including country as a predictor did not alter any conclusions. Hence, we present analyses of the dataset as a whole.

## 3 Results

### 3.1 Descriptive statistics

Descriptive statistics for key variables are shown in Table 1.

**Table 1:** Descriptive statistics.

	Overall (N = 484)
**Age**	
Mean (SD) [Min, Max]	41.9 (10.5) [25, 76]
N	484
**Gender**	
Woman	244 (50.4 %)
Man	234 (48.3 %)
PNTS or self-describe	6 (1.2 %)
N	484
**Average income**	
Mean (SD) [Min, Max]	3430 (1720) [515, 11400]
N_individuals_, N_reports_	484, 4874
**Income volatility**	
Mean (SD) [Min, Max]	770 (1200) [0, 7780]
N	484
**Self-rated health**	
Mean (SD) [Min, Max]	6.10 (1.67) [1.17, 9.00]
N_individuals_, N_reports_	484, 4816
**Anxiety-depression**	
Mean (SD) [Min, Max]	5.85 (4.75) [0, 22.5]
N_individuals_, N_reports_	484, 4836

Self-rated health and anxiety-depression were correlated at *r* = −0.67.

### 3.2 Associations between volatility and health

**Fig. 2:**
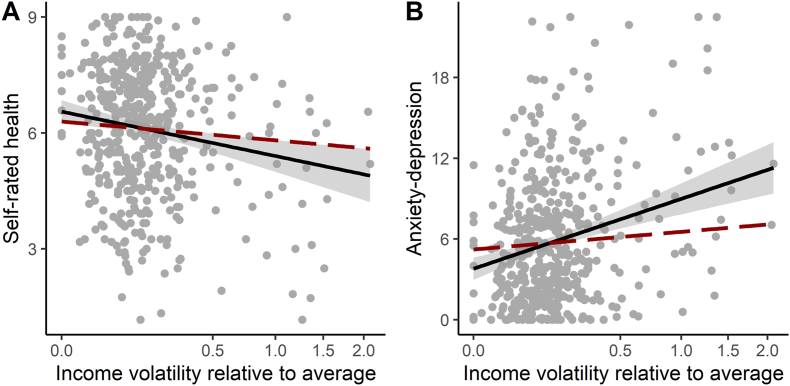
Observed associations between income volatility and self-rated health (A) and anxiety-depression (B). Income volatility is here expressed relative to average income, so a value of 0.5 means that the month-to-month standard deviation of a person's income is equal to half their average income. The solid line shows the observed associations, whilst the dashed lines show the associations that would be predicted based on concavity effects alone. Grey shading indicates the 95 % confidence interval.

Income volatility was a significant predictor of both average self-rated health (negative), and anxiety-depression (positive), after controlling for average income, age and gender (Table 2, Fig. 2). The variance explained by income volatility (partial $\eta^{2}$) was similar to that explained by average income for self-rated health (average income: 0.034; volatility: 0.025), and greater than that explained by average income for anxiety-depression (average income: 0.022; volatility: 0.049).

**Table 2:** Summary of regression models predicting self-rated health and anxiety-depression.

Predictors	Self-rated health			Anxiety-depression		
	Estimates	CI	p	Estimates	CI	p
(Intercept)	1.14	−1.09–3.36	0.317	22.83	16.71–28.94	<0.001
Average income	0.74	0.45–1.03	<0.001	−1.95	−2.74–−1.16	<0.001
Income volatility	−0.18	−0.29–−0.07	0.002	0.64	0.33–0.95	<0.001
Age	−0.00	−0.01–0.01	0.933	−0.11	−0.15–−0.07	<0.001
Gender (man)	0.27	−0.02–0.56	0.069	−0.90	−1.71–−0.10	0.028
Gender (PNTS or self-describe)	−0.54	−2.40–1.32	0.567	1.86	−3.24–6.97	0.473

Observations	478			478		
R^2^/R^2^ adjusted	0.063/0.053			0.133/0.123

### 3.3 Concavity-predicted health variables

**Fig. 3:**
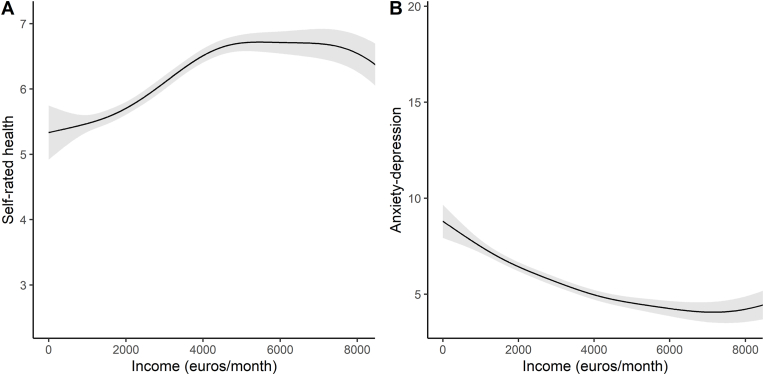
Visualization of best-fitting GAMs for self-rated health (A) and anxiety-depression (B) against income. Shading shows 95 % confidence interval. Incomes are truncated at the 95th percentile (around 8000 euros) as beyond this point the shape of the GAM is affected by a small number of very high incomes. All income values are used in the analysis.

The shapes of the fitted GAMs are shown in Fig. 3. To establish concavity, we evaluated the first and second derivatives of each GAM at each income value observed in the dataset, and took the mean of these. The GAM for self-rated health on income had a positive first derivative on average (0.00024) and a negative second derivative on average (−0.18). The GAM for anxiety-depression had a negative first derivative on average (−0.00067) and a positive second derivative on average (0.23). Thus, the GAMs were, overall, upward concave and downward concave as expected.

We used the predicted values from the GAMs to compute, for each participant, the average self-rated health and average anxiety-depression that they would be expected to have due to concavity effects alone. Income volatility was significantly associated with concavity-predicted self-rated health and concavity-predicted anxiety-depression, as expected (Table 3). However, the associations were much weaker than the observed associations between income volatility and the actual outcomes. The coefficient of the actual association between income volatility and self-rated health was −0.18 whereas the concavity-predicted association was −0.06. For anxiety-depression, the observed association was 0.64 whereas the concavity-predicted association was 0.16. Thus, the observed associations were respectively 3 times and 4 times stronger than expected from concavity effects alone.

**Table 3:** Summary of regression models where the outcome was concavity-predicted self-rated health and concavity-predicted anxiety-depression.

Predictors	Self-rated health			Anxiety-depression		
	Estimates	CI	p	Estimates	CI	p
(Intercept)	0.99	0.66–1.31	<0.001	18.45	17.83–19.07	<0.001
Average income	0.70	0.65–0.74	<0.001	−1.73	−1.81–−1.65	<0.001
Income volatility	−0.06	−0.08–−0.05	<0.001	0.16	0.13–0.19	<0.001
Age	−0.00	−0.00–−0.00	0.027	0.00	0.00–0.01	0.024
Gender (man)	−0.01	−0.05–0.04	0.736	0.01	−0.07–0.09	0.778
Gender (PNTS or self-describe)	0.01	−0.26–0.28	0.935	−0.04	−0.56–0.48	0.879

Observations	478			478		
R^2^/R^2^ adjusted	0.703/0.700			0.800/0.798		

### 3.4 Multilevel approach

Using the data at the month level, we fitted multilevel GAMs predicting self-rated health and anxiety-depression from a smooth function of income from that month (a level 1 variable), plus age and gender (level 2 variables), with a random intercept for participant.

In a second step, we added (logged) income volatility as an additional level 2 predictor variable. In these models, income volatility was a significant negative predictor of self-rated health (B = −0.11, 95 % CI -0.22 to −0.01, p = 0.031) and a significant positive predictor of anxiety-depression (B = 0.44, 95 % CI 0.15 to 0.72, p = 0.003). Full model tables for the multilevel models are provided in the Supporting Materials (Appendix A).

### 3.5 Counterfactual analysis

**Fig. 4:**
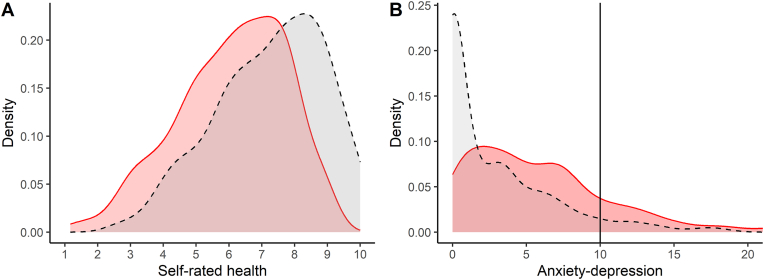
Distribution density plots for the health variables as they are (solid lines and red shading), and under the counterfactual scenario where income volatility is zero for every person. (A) Self-rated health. (B) Anxiety-depression. The vertical line on panel B reflects the cutoff for clinical concern, 10, for both component measures.

We compared the predicted values for the health outcome variables under the actual values of income volatility, and a counterfactual scenario where all individuals had an income volatility of zero. The results are shown in Fig. 4. The change in self-rated health under the zero-volatility counterfactual equates to a Hedge's *g* of −0.69 (95 % CI -0.82 to −0.56). For anxiety-depression, it equates to 0.7 (95 % CI 0.57 to 0.83).

We performed the same exercise for the constituent measures of the anxiety-depression score (i.e. GAD-7 and PHQ-8) separately. This allowed us to compare the percentage of people averaging above 10 (the cutoff for clinical concern) in the actual data and under the zero-volatility counterfactual. For GAD-7, the percentage was 18.5 in actuality, and 7.7 under the zero-volatility scenario. For PHQ-8, the respective percentages were 17.8 and 7.1.

## 4 Discussion

Using data from the CCLS, a small panel from the UK and France, we found clear evidence that month-to-month income volatility was related to two health measures, self-rated general health, and a measure of anxiety and depression symptoms, after controlling for the level of income. Higher volatility was associated with worse self-rated health, and more symptoms of anxiety and depression. In this dataset, income volatility accounted for a comparable amount of variation in health outcomes as the level of income: slightly less for self-rated health, slightly more for anxiety-depression. Moreover, the predicted change at the population level in the counterfactual where income volatility was reduced to zero for everyone was large: around seven tenths of a standard deviation improvement in both health variables; and the number of individuals above the clinical concern cutoff and the anxiety and depression symptom scales reduced to less than half its observed value.

Our findings are consistent with the conclusions of previous investigations of income volatility (Adeline et al., 2019; Grasset et al., 2019; Rohde et al., 2016; Sayre, 2023). However, those investigations measured fluctuations over the timescale of either days or years, whereas key outgoings for many people fall over a monthly cycle. Thus, month-to-month volatility, which we studied here, may be particularly relevant for mental and physical health, and provides a particularly clear demonstration. The fact that studies measuring volatility at several different timescales have come to similar conclusions suggest that the association between volatility and health might be quite general, whichever timescale is chosen.

Some association between volatility and health is to be expected because of concavity effects. Since the association between income and health is usually concave, a downward fluctuation of a given magnitude is worse for health than the equivalent upward fluctuation. We showed that the associations between income and health variables were indeed concave in the present data. We computed the strength of associations between income volatility and health that were expected solely due to concavity effects. The observed associations were 3 (self-rated health) and 4 (anxiety-depression) times stronger than this. This is a sufficient margin to make us confident that the difference is not just due to measurement error, or failure to adequately model the concavity in the data. This is especially so since we used GAMs, a flexible non-linear modelling approach that use the data to determine the shape of the association. We explored several modelling approaches, using both single-level and multilevel models, and both GAMs and a log-linear regression to capture the concavity, and consistently concluded that volatility mattered independently of the level of income and more than expected through concavity effects.

Our results, and those of previous studies of income volatility, are consistent with three pathways: income volatility has a negative effect on health (causation); poor health induces income volatility (reverse causation); or income volatility is correlated with some health-damaging third variable not included in our models (third variable). Our results are consistent with the first pathway being important, but present research design cannot exclude contributions from the other two. To identify the direction of causation, long time series (or natural experiments) would be needed, in which people experience changes in volatility, or changes in health, to establish the extent to which change in each one leads to change in the other. The twelve-month time series of the CCLS is not sufficiently long to clearly identify changes in income volatility.

As for third-variable explanations, jobs that produce volatile income may also tend to be worse for other reasons: lower autonomy and control, worse working relationships, or less regular hours. The CCLS does not have measures of these variables, and thus it remains a limitation of our study that we cannot exclude that some combination of them explains the volatility associations. This highlights the need for similar investigations in larger cohorts where more variables may be measured. The omission of important third variables is particularly relevant to the counterfactual analysis shown in Fig. 4. This analysis assumes that the observed associations between income volatility and health, after controlling for average income, age and gender, entirely reflect the causal impact of volatility, and not third variables that would be unaffected by intervening on volatility. We thus offer this counterfactual analysis for illustrative purposes rather than as a quantitative causal prediction about what would happen if volatility were eliminated.

These interpretive limitations acknowledged, it remains plausible that causation is important, and volatility itself is detrimental to health. That is, there is a health cost of one's financial situation fluctuating, above and beyond the sum of the situations through which one passes. Volatility is likely to create uncertainty, for example about whether key bills can be paid. Volatility and uncertainty are not the same thing. A job could in principle generate income streams that varied from month to month, but in a predictable way that was known in advance. In this case there would be volatility but no uncertainty. However, we suspect that in practice, those with more volatile income streams also experience more uncertainty. Uncertainty generates psychological stress (Berker et al., 2016; Peters et al., 2017), which is closely linked to poor mental and physical health. This remains an interpretation, since we have no direct measure of perceived stress in our study. Again, this is a limitation that can be overcome in future research that investigates the mediating role of stress.

The samples taking part in the CCLS were not nationally representative. Participants were disproportionately drawn from lower income groups, and, since they were enrolled with market research providers to do surveys for recompense, may have had unusually high levels of income volatility. These limitations primarily affect inferences about absolute levels of income volatility or health in the UK and France. Since our main conclusions concern not the levels of either variable, but the relationships between them, they are relatively robust, but it is a priority to investigate the health consequences of income volatility in other panel datasets. Income was self-reported. Wealth was not measured, but is potentially important since savings can buffer the impact of volatility in incomes.

Our findings have implications for population health, public policy, and institutional design. Income volatility is common in contemporary affluent societies, and the findings imply that there is a burden of preventable morbidity directly attributable to this. This implies that there are health gains from all kinds of institutional reforms that either reduce volatility, or blunt its impact. Reforms that reduce volatility include, for example, changes to labour market organization or to welfare regimes. For example, Johnson et al. (2025) recently argued that introducing an unconditional universal basic income coupled with progressive taxation would have two distinct effects. It would be redistributive: those with the lowest incomes would gain the most. Critically from the current perspective, it would also reduce the volatility of income streams, since it would truncate the fluctuations. The present findings suggest that this latter consequence would have a positive effect on population health. Yet, attempts to model the health impact of introducing basic income (e.g. Reed et al., 2024) have only modelled the health gain attributable to the redistributive effect. This suggests that they may substantially underestimate the potential health benefits.

Reforms that blunt the impact of volatility are those that decommodify the meeting of basic needs. If people can meet basic needs independently of the ups and downs of their incomes, the damaging potential of those ups and downs is mitigated. We suggest that cost-benefit analyses of policy and institutional changes should include the effects attributable to any income volatility they prevent, introduce, or mitigate, as well as just their effects of the population distribution of levels of income.

## CRediT authorship contribution statement

**Daniel Nettle:** Writing – review & editing, Writing – original draft, Visualization, Software, Investigation, Formal analysis, Data curation, Conceptualization. **Coralie Chevallier:** Writing – review & editing, Project administration, Funding acquisition, Conceptualization. **Kate E. Pickett:** Writing – review & editing, Funding acquisition, Conceptualization. **Matthew T. Johnson:** Writing – review & editing, Conceptualization. **Elliott A. Johnson:** Writing – review & editing, Conceptualization. **Melissa Bateson:** Writing – review & editing, Writing – original draft, Visualization, Validation, Formal analysis, Conceptualization.

## Author declaration

The authors declare that they have no competing interests.

## Ethical statement

The Changing Cost of Living Study was approved by the Newcastle University Research Ethics Committee (REC). The present paper reports secondary analysis of the anonmyised data.

## Financial disclosure

The authors declare that they have no financial interests that could have appeared to influence the work reported in this paper. No external funding was received for this study.

## Declaration of competing interest

The authors declare that they have no competing interests. No external funding was received for this study.

## Data Availability

Data and code are available here: https://osf.io/49w3z/
