# Supplementary material for: Why is income volatility associated with poor health? Longitudinal evidence from the UK and France
Source: SSM Popul Health. 2025 Oct 6;32:101869. doi: 10.1016/j.ssmph.2025.101869 (PMC12547960; doi:10.1016/j.ssmph.2025.101869)
Supplement: Multimedia component 1 [file mmc1.pdf]

Supporting information for: Why is income volatility associated with poor health? Longitudinal evidence from the UK and France

Alternative specification using regression on the logarithm of income

As an alternative to the GAMs for characterizing concavity in the income-health relationships, we used regression models of the outcome variables on the logarithm of income. We first established that models using log(income) fit the data better than those using linear income. This was indeed the case: for both self-rated health and anxiety-depression, the root mean squared error (RMSE) was lower for a model predicting the outcome from the log of income than from linear income (self-rated health: log 1.94 vs. linear 1.95; anxiety-depression: log 5.19 vs. linear 5.24). The regression equation $Y = \beta_0 + \beta_1 \log(X)$ has the second derivative $-\frac{\beta_1}{X^2}$. The sign of this is the opposite of the sign of β_1 for all positive values of X. β_1 was 0.46 for self-rated health and -1.26 for anxiety-depression. Thus, as required, the relationship between income and self-rated health was downwards concave for self-rated health and upwards concave for the association between income and anxiety-depression.

Paralleling the analysis in the main paper, we used the parameter estimates from the logarithmic model to derive the equation linking log income to the health variables, and hence to calculate the health status each participant should have been expected to have in each month given their income that month. We averaged these variables across the months to give, for each participant, the average self-rated health and average anxiety-depression that they would be expected to have due to concavity effects alone.

Income volatility was significantly associated with predicted self-rated health and predicted anxiety-depression, as expected (table S1). The logarithmic-model based predicted associations were around half as strong as strong as those reported in the main paper using GAMs (self-rated health: GAM -0.06 vs. logarithmic -0.03; anxiety-depression: GAM 0.16 versus logarithmic 0.08). These were much weaker that the observed associations between income volatility and the outcome variables which, for recall, were -0.18 for self-rated health and 0.08 for anxiety-depression. Thus, modelling the concavity using logarithmic models leads to the conclusion that the associations between income volatility and the outcomes are respectively 6 and 8 times greater than could be predicted based on concavity alone (versus 3 and 4 using GAMs). The GAM result is the more robust, as well as the more conservative, since GAMs capture the observed concavity more precisely, as evidenced by their better fit than a log model (RMSE for GAM versus log models: self-rated health: GAM 1.91, log 1.94; anxiety-depression: GAM 5.15; log 5.19).

Table S1: Summary of regression models where the outcome was concavity-predicted general health and concavity-predicted anxiety-depression, using the logarithmic regression approach.

	Self-rated health			Anxiety-depression		
Predictors	*Estimates*	*CI*	*p*	*Estimates*	*CI*	*p*
(Intercept)	2.28	2.14 – 2.42	**<0.001**	16.16	15.78 – 16.53	**<0.001**
Average income	0.50	0.49 – 0.52	**<0.001**	-1.37	-1.42 – -1.32	**<0.001**
Income volatility	-0.03	-0.04 – -0.02	**<0.001**	0.08	0.07 – 0.10	**<0.001**
Age	-0.00	-0.00 – 0.00	0.381	0.00	-0.00 – 0.00	0.381
Gender (Man)	-0.01	-0.02 – 0.01	0.551	0.01	-0.03 – 0.06	0.551
Gender (PNTS or self-describe)	0.02	-0.10 – 0.13	0.791	-0.04	-0.35 – 0.27	0.791
Observations	478			478		
R^2 / R^2 adjusted	0.877 / 0.876			0.877 / 0.876		

Model tables for multilevel approach

Table S2: Results of multilevel models (see main paper for details).

	Self-rated health			Anxiety-depression		
Predictors	*Estimates*	*CI*	*p*	*Estimates*	*CI*	*p*
(Intercept)	6.66	5.74 – 7.58	**<0.001**	8.40	5.89 – 10.91	**<0.001**
Income volatility	-0.11	-0.22 – -0.01	**0.031**	0.44	0.15 – 0.72	**0.003**
Age	-0.00	-0.01 – 0.01	0.940	-0.11	-0.15 – -0.07	**<0.001**
Gender (man)	0.28	-0.01 – 0.57	0.060	-0.96	-1.77 – -0.15	**0.020**
Gender (PNTS or self-describe)	-0.63	-2.17 – 0.90	0.417	0.80	-3.32 – 4.92	0.704
Income (smooth term)			**<0.001**			**<0.001**
Observations	4784			4784		
Individuals	481			481		
Variances:						
Individual	2.51			19.46		
Residual	1.13			4.74		
R^2:						
Marginal	0.02			0.09		
Conditional	0.69			0.82		
